# Streamlined detection of Nipah virus antibodies using a split NanoLuc biosensor

**DOI:** 10.1080/22221751.2024.2398640

**Published:** 2024-08-28

**Authors:** Éric Bergeron, Cheng-Feng Chiang, Michael K. Lo, Elif Karaaslan, Syed Moinuddin Satter, Mohammed Ziaur Rahman, Mohammad Enayet Hossain, Wasik Rahman Aquib, Dewan Imtiaz Rahman, Subyeta Binte Sarwar, Joel M. Montgomery, John D. Klena, Christina F. Spiropoulou

**Affiliations:** a Viral Special Pathogens Branch, Division of High-Consequence Pathogens and Pathology, National Center for Emerging and Zoonotic Infectious Diseases, Centers for Disease Control and Prevention, Atlanta, USA; b Department of Pharmaceutical and Biomedical Sciences, University of Georgia, Athens, USA; c icddr,b, Dhaka, Bangladesh

**Keywords:** Nipah virus, serology, disease surveillance, antibodies, paramyxoviridae

## Abstract

Nipah virus (NiV) is an emerging zoonotic RNA virus that can cause fatal respiratory and neurological diseases in animals and humans. Accurate NiV diagnostics and surveillance tools are crucial for the identification of acute and resolved infections and to improve our understanding of NiV transmission and circulation. Here, we have developed and validated a split NanoLuc luciferase NiV glycoprotein (G) biosensor for detecting antibodies in clinical and animal samples. This assay is performed by simply mixing reagents and measuring luminescence, which depends on the complementation of the split NanoLuc luciferase G biosensor following its binding to antibodies. This anti-NiV-G “mix-and-read” assay was validated using the WHO's first international standard for anti-NiV antibodies and more than 700 serum samples from the NiV-endemic country of Bangladesh. Anti-NiV antibodies from survivors persisted for at least 8 years according to both ⍺NiV-G mix-and-read and NiV neutralization assays. The ⍺NiV-G mix-and-read assay sensitivity (98.6%) and specificity (100%) were comparable to anti-NiV IgG ELISA performance but failed to detect anti-NiV antibodies in samples collected less than a week following the appearance of symptoms. Overall, the anti-NiV-G biosensor represents a simple, fast, and reliable tool that could support the expansion of NiV surveillance and retrospective outbreak investigations.

## Introduction

Emerging infectious diseases represent a global threat to public health for which diagnostic and surveillance tools are needed for improved outbreak preparedness and response. Henipaviruses, such as Nipah virus (NiV) and Hendra virus (HeV), are paramyxoviruses that were first recognized in the 1990s to cause severe and highly lethal infections in horses, pigs, and humans [1–3]. NiV was discovered in 1998 in Malaysia and Singapore during an outbreak linked to infected pigs [3]; *Pteropus* bats, commonly called flying foxes, serve as the viral reservoir and incidental infection animal hosts [4–6]. In Malaysia and Singapore, human NiV infections had a reported case fatality rate (CFR) of around 40% [1], with primarily neurological symptoms. Contrastingly, in India and Bangladesh [7], the infection has presented principally as a respiratory disease, with several instances of human-to-human transmission [8,9] and a CFR of approximately 70%. In addition, rare HeV human infections were reported in Australia and also often led to fatal respiratory and neurological conditions [2]. More recently, additional henipaviruses have been identified in Africa and Asia, including Ghana virus, Cedar virus (Australia), Gamak virus (South Korea), Langya virus, and Mojiang virus (both China) [10], and some of these viruses have been associated with febrile illness in humans.

Serum analyses for NiV-specific antibodies play a crucial role in diagnostics, epidemiology, and monitoring of virus circulation in both humans and animals [11]. Enzyme-linked immunosorbent assays (ELISAs) are typically used to identify NiV-specific IgM and IgG antibodies. The presence of anti-NiV IgM generally indicates that an individual either has an acute or recent infection, while in the absence of symptoms, anti-NiV IgG generally indicates previous exposure. These tests are valuable for diagnosing NiV infection, evaluating infection burden of infection, and developing targeted public health interventions. However, commonly used ELISAs or Luminex bead-based assays typically require lengthy, complex protocols and highly trained personnel, limiting their use to specialized laboratories. Although NiV neutralization test can serve as an alternative to ELISA [11], it requires high-containment laboratory facilities not available in many endemic countries such as Bangladesh.

Since NiV is a zoonotic virus, seroprevalence studies should ideally be conducted in both animals and humans to identify potential subclinical infections, as recently reported in India [12], and larger segments of the population at high risk of exposure to NiV. This type of work is critical, but it requires properly validated serological assays to accurately estimate the infection prevalence rate to guide interventions aimed at reducing exposure risk. For NiV, the characterization and validation of serological assays has been particularly challenging, mainly due to difficulties in obtaining clinical specimens from confirmed cases that survive this highly lethal infection. As a result, the interpretation of NiV serology is limited, as current assays are typically used with experimental or scant clinical samples.

Innovative technologies like split nanoluciferase (NanoLuc) have recently emerged as much more simple alternatives to traditional diagnostic methods that do not compromise performance [13–16], making them suitable for identifying infections in a point-of-care format [17]. To detect SARS-CoV-2 antibodies, serum or plasma samples were combined with the spike receptor binding domain (RBD) SmBiT and LgBiT fusion proteins; the emitted light is measured as NanoLuc is reconstituted, dependent on the presence of antigen-specific antibodies [14,15]. Here, we detail the development and validation of a fast and simple split NanoLuc biosensor detecting NiV antibodies in serum or plasma clinical samples from Bangladesh, and demonstrate that this straightforward assay is an ideal tool for serological NiV surveillance.

## Materials and methods

### Ethics statements

For human samples from Bangladesh, the institutional research protocol PR-2005-026 was reviewed and approved by the ethical review board of International Centre for Diarrheal Disease Research, Bangladesh (icddr,b). All the respondents were requested to provide written informed consent/assent to participate in this study. If the patient was unable to provide informed consent or was less than 18 years of age, the legal guardian/caregiver were requested to provide information for the questionnaire and consent on behalf of the patient. Assent was taken from children aged between 11–18 years.

Human sera from resident of the United States of America (USA) were purchased from BioVT and human and animal control sample from Malaysia is a residual diagnostic sample that was collected as part the Nipah virus outbreak response in 1998.

#### Clinical samples and virus inactivation

Samples collected in Bangladesh from human patients with suspected infections during NiV outbreaks from 2012 to 2021 and residual sample from 1998 Malaysia outbreak were analyzed in this study. All serum and plasma samples were inactivated by gamma irradiation with 5 × 10^6^ rad before analysis using ELISAs and mix-and-read (MR) assays. Human sera from the USA were heat-inactivated at 56°C for 30 min.

#### Virus stocks, cell lysates, slurries, and antibodies

NiV strain SPB199901924, Malaysian prototype, was isolated from the cerebrospinal fluid of a patient who died during the 1998 outbreak in Malaysia. Viruses were inoculated into Vero-E6 cells and propagated until cytopathic effect (CPE) was observed by immunofluorescent assays (IFA) before harvest. For making cell slurries, the scraped cells and their culture media were collected and pooled. Otherwise, the scraped cells were centrifuged and washed before lysis in borate saline containing 1% Triton X-100 (pH 9). Subsequently, the slurries were frozen at – 70°C and gamma-irradiated at 5 × 10^6^ rad before sonication. An additional centrifugation step was required to obtain cell lysates free of insoluble material. Aliquots of cell slurries or lysates were lyophilized in small quantities for long-term storage at – 20°C before use. Control lysates or slurries of uninfected Vero-E6 cells were prepared in the same matter. Anti-NiV, anti-HeV, anti-Sosuga virus, anti-parainfluenza virus 1, and anti-parainfluenza virus 5 hyperimmune mouse ascitic fluid (HMAF) were produced using a previously described protocol [18]. Monoclonal antibodies (mAbs) 12B2, nAH1.3, and 7B7 were purchased from Absolute Antibody Ltd. The WHO First International Standard for NiV antibodies for binding and neutralization assays (human sera) 22/130_BA and 22/130_NT were kindly provided by the National Institute for Biological Standards and Control (Potters Bar, Hertfordshire, United Kingdom) [19].

#### Indirect IgG ELISA

Lyophilized NiV-infected or control Vero-E6 cell lysates were rehydrated with water and coated overnight at 4°C at 1:1000 in phosphate buffered saline (PBS). Serum samples from NiV patients or serum controls diluted 4-fold (1:100–1:6400) in serum diluent (5% skim milk in PBS, 0.1% Tween-20 [pH 7.4]) were incubated for 1 h at 37°C and then washed with PBS containing 0.1% Tween-20 (PBST; also included in the subsequent steps). Mouse anti-human IgG Fcγ HRP conjugate (Jackson ImmunoResearch, West Grove, PA) was subsequently added and incubated at 1:4000 for 1 h at 37°C. The peroxidase reaction was developed with 2,2′-azino-bis-3-ethylbenzthiazoline-6-sulphonic acid (ABTS) substrate system (KPL) for 30 min, and optical density (OD) was read at 410 nm. The OD values were subtracted from the background value of control uninfected Vero-E6 cell lysates to calculate adjusted OD. Test samples were considered positive if their mean adjusted OD was greater than the mean adjusted OD of blank samples (wells without specimen or serum controls) plus 3 times the standard deviation (indicated as the signal cutoff value), and the mean sum OD (calculated from the adjusted OD) of blanks plus 3 times of their standard deviation (indicated as the Sum OD cutoff value). The assay signal and sum OD cut-off values were determined as 0.2 and 0.95, respectively, as described above.

#### IgM-capture ELISA

IgM against NiV in human sera was detected by ELISA modified from methods previously described for Ebola virus [20]. This IgM-capture assay uses goat antibodies against the Mu chain of human IgM (KPL; solubilized to 1 mg/mL in 50% glycerol in water) to capture IgM in serum samples from NiV patients, diluted serially 4-fold; the reaction was incubated for 1 h at 37°C. Lyophilized slurries from NiV-infected cells were rehydrated in water with 0.1% Tween-20, then diluted 6-fold in serum diluent and used as antigen for the bound IgM. Normal human serum and lyophilized, purified native human IgM (Bio-Rad,), solubilized to 1 mg/mL of 50% glycerol (diluted in PBS), were diluted 1:25 and 1:500, respectively, in the cell slurry to occupy the unbound anti-human IgM Mu and reduce the assay’s background. Anti-NiV HMAF (1:4000) was produced and used as the detecting antibody. Subsequently, goat anti-mouse HRP conjugate (Thermo Scientific, Waltham, MA) diluted 1:8000 in serum diluent was used as the secondary antibody for signal amplification, as described above. Slurries of uninfected Vero-E6 cells were used as the assay control for detecting non-specific binding signal from human sera. The assay signal and sum OD cut-off values were determined as 0.1 and 0.45, respectively, as described above.

#### Niv G biosensors production and purification

NiV G biosensor sequences were synthesized by Twist Biosciences and cloned into a mammalian expression vector pEEV-Puro [15]. Biosensors comprise the sequence of NiV G strain Malaysia (residues 171–602; GenBank accession no. AAK50545.1). An mouse IgG kappa chain signal peptide and 8×His tag were included at the N-terminus of all the constructs to allow secretion into the cell supernatant and purification by immobilized metal affinity chromatography (IMAC). The NanoBiT fragments, LgBiT and SmBiT, were fused to either the N – or C – terminal end of the soluble head domain of G, with a flexible linker between the two. These proteins were produced in Expi293F cells (Thermo Fisher Scientific) cultured in Expi293 expression medium via transient transfection using FectoPro transfection reagent following the manufacturer’s guidelines (Polypus). Specifically, the cells were transfected with 0.8 μg plasmid/mL of culture using 1.5 μL of transfection reagent per μg of plasmid. Four to six days post-transfection, the culture supernatants were clarified, filtered, and applied to the HisTrap Excel column, followed by size exclusion chromatography equilibrated with PBS (Superdex 200 increase 16/600 GL, Cytiva). Proteins were quantified and stored at – 80°C.

#### Anti-NiV-G mix-and-read assay

A 15 nM equimolar solution of anti-NiV-G-LgBiT and NiV-SmBiT biosensors was prepared in PBS (pH 7.4) supplemented with 0.2% BSA and 0.05% Tween-20. Next, 15 µL αNiV-G biosensor solution was added to a half-area white 96-well microplate (Corning), followed by the addition of 15 µL of plasma or serum and a 20 min incubation at ambient temperature. After incubation with the biosensor, 30 µL of NanoGlo® Assay Reagent (Promega) was added to the well and incubated for 10 min before reading with a Synergy Neo2 instrument with a photomultiplier tube (PMT), gain of 130 and a read time 1 s. Control sera consisted of normal human serum (BioVT) and human NiV-positive IgM ^+ ^IgG^+^ (VSPB: SPR611) diluted 1:5 in normal human serum. Signal-to-noise ratios (SNR) for each experimental sample (no replicates) were obtained by dividing the relative fluorescence units (RLU) from the experimental samples by the mean RLU obtained from normal human serum triplicate reactions.

#### Virus neutralization assay

Each serum/plasma sample was initially diluted either 1:5 or 1:10 with DMEM-10% FBS, and then serially diluted 2-fold for 8 dilutions in a U-bottom polypropylene plate. An equivalent volume of NiV (Bangladesh strain) inoculum (∼ 125 TCID_50_) was then added to each dilution of plasma, mixed, incubated at 37°C for 30 min, and then dispensed into 96 well plates containing 10,000 Vero cells per well. On day 4 post infection, wells were scored for the presence or absence of CPE. Final sample dilutions ranged between 1:20–1:5120. The lowest dilution at which all replicate wells (duplicate or quadruplicate) were negative for CPE was considered the virus neutralization titer (VNT_100_). Rabbit polyclonal serum raised against the soluble HeV G glycoprotein was used as a positive control for virus neutralization.

#### Data analysis

Receiver operating characteristics (ROC) analyses and Pearson correlation coefficients were calculated using GraphPad Prism v.10.2.

## Results

### Development of split NanoLuc biosensors to detect anti-NiV antibodies

**Figure 1.**
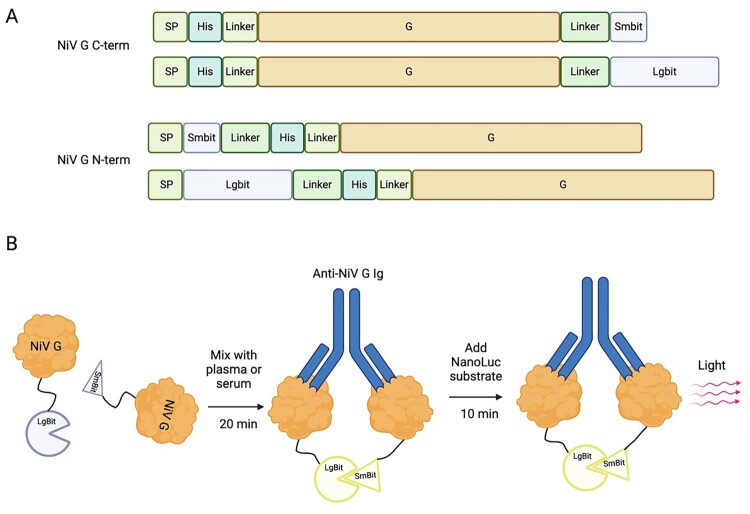
αNiV-G biosensor design and assay principle for detecting anti-NiV G antibodies. (A) Nipah virus (NiV) G signal peptide was replaced by IgG-kappa chain signal peptide, and a histidine tag was included for purification from the cell supernatant. The SmBiT and LgBiT NanoLuc fragments were fused to either the N – or C – terminus of a soluble form of G from NiV strain Malaysia (amino acids 136–556). (B) Activation of αNiV-G biosensors by human convalescent serum and anti-G monoclonal antibodies (mAbs). Simultaneous binding of NiV-G-SmBiT and NiV-G-LgBiT by specific antibody-mediated complementation of SmBiT and LgBiT results in functional NanoLuc luciferase, which produces light after the addition of NanoLuc substrate furimazine.

**Figure 2.**
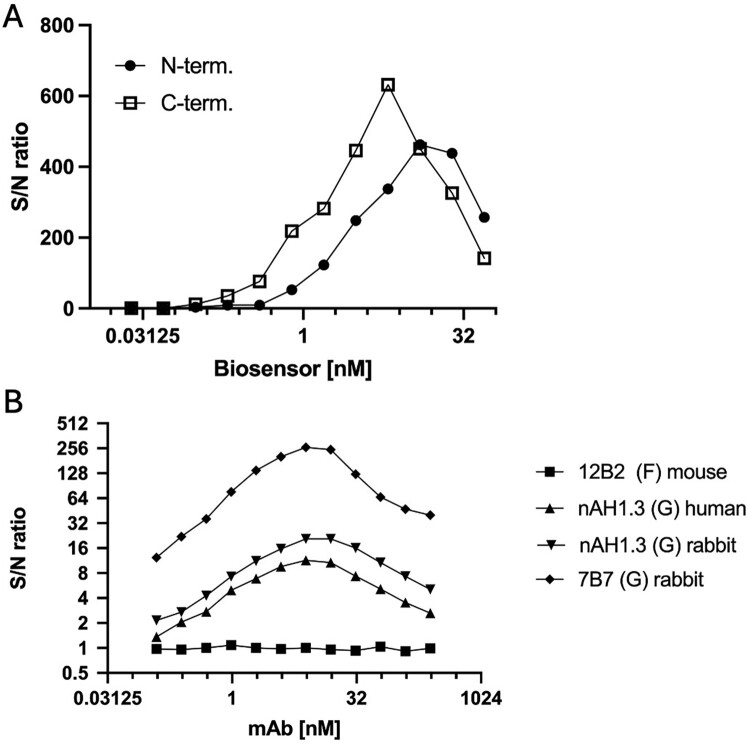
Optimization of G-biosensor concentration and its specific activation by anti-G monoclonal antibodies. (A) Human serum from NiV-confirmed cases was incubated with increasing concentrations of G-biosensor N – and C-terminal pairs to determine the maximal signal-to-noise ratio (S/N ratio; Y-axis). The concentrations of the G-biosensor pairs (X-axis) are actual reaction concentrations before the addition of NanoLuc substrate. (B) G C-terminal pair reaction concentration was fixed at 7.5 nM and incubated with variable concentrations of anti-NiV mAbs.

Various source antigens have been used in anti-NiV ELISAs to detect antibodies, including NiV-infected cell lysate and recombinant glycoprotein (G), fusion protein (F), and nucleocapsid (N) [21–25]. The WHO has recently set an international global standard for quantifying and normalizing the results from anti-G serological assays [19]. To utilize this established standard, we created G biosensors by adding LgBiT and SmBiT fragments of NanoLuc luciferase to the N – or C-terminal ends of G from NiV-Malaysia (Figure 1A), the membrane-proximal and transmembrane anchor were deleted to allow secretion into the supernatant. Following transfection of Expi293 cells, the histidine-tagged soluble G constructs were isolated through affinity and size exclusion chromatography to produce highly purified biosensor G-SmBiT and G-LgBiT pairs. Both arms of the anti-NiV G antibodies must bind simultaneously to the SmBiT and LgBiT G-fusion protein for NanoLuc reconstitution (Figure 1B). We initially assessed the performance of N – and C-terminal biosensor pairs at different concentrations using human serum from a recovered patient from the Malaysia NiV outbreak, comparing it with normal human serum to determine the optimal signal-to-noise (SNR) defined as RLU NiV serum/RLU normal serum. C-terminal G fusion produced the best SNR at a final concentration of 6.25 nM, while the N-terminal fusion yielded a lower SNR at a higher concentration (Figure 2A). Given its superior performance in terms of SNR and concentration, further characterization and experiments were limited to the C-terminal G-fused biosensor, termed anti-NiV-G (αNiV-G) biosensor.

NiV G monoclonal antibodies were then assessed for their capability to activate the αNiV-G biosensor. αNiV-G concentration remained constant at 7.5 nM during incubation with varying mAb concentrations; an anti-NiV F mAb was included as a negative control. In agreement with the binding model requiring the biosensor to be activated by the antibody (Figure 1B), maximum SNR (i.e highest signal measured when antibody concentration varies) was achieved for binding mAbs (7B7 and nAH1.3) at approximately 7.8 nM, closely matching the αNiV-G biosensor concentration of 7.5 nM. The maximum SNR for rabbit mAb nAH1.3 was around 13 times lower than that of rabbit 7B7. Maximum SNR was less than ∼1.8 times higher than that of human nAH1.3, a mAb sharing the same G binding region but differing in the species-specific constant mAb region, which is not involved in G binding. Overall, these data confirm that the NiV-G C-terminal fusion biosensor pair is superior at detecting antibodies, and that specific anti-G mAbs from human or rabbit mAb can maximally activate the G-biosensor at the expected binding stoichiometry.

### Correlation of G-biosensor signal with ELISA and virus neutralization assays

To create the first international anti-NiV antibody standard, the WHO recently published the results from serological assays performed by 18 independent laboratories using a set of 10 standard samples (NV1-NV10). These results were used to create the first international anti-NiV antibody standard [19]. Although the G biosensor was not designed for the precise quantification of antibodies, as this would require additional dilution steps and validation, we compared how the SNR values matched those from ELISA and neutralization assays performed elsewhere using the same standard samples [19]. The concentration of the international standard (sample NV1, Supplementary Table 1) was established at 1000 international units/mL (IU/mL) and was used to compare the G-biosensor SNR and results from quantitative anti-G ELISAs from 4 independent laboratories. In addition, CDC’s non-quantitative ELISA, which uses NiV-infected cells, was included to further compare the methods results. The biosensor SNR values of standard samples (NV2-NV9) were normalized to the international standard sample (NV1) defined as 1000 IU/mL [sample IU/mL  = (1000 IU/mL*sample SNR)/NV1 international standard SNR]. In agreement with other anti-G-ELISA published assays, NV7 and NV9 were found negative using CDC ELISA, and normalized SNR values of these negative samples were equal to 2 IU/mL, while positive samples ranged 81–1000 IU/mL. The correlation levels were evaluated by determining the Pearson correlation coefficient (r) between anti-GELISAs and G-biosensor results. A significant correlation was observed with all quantitative anti-G ELISAs. There was a strong correlation between the G-biosensor readings and the NiV Bangladesh anti-G ELISAs, with correlation coefficients exceeding 0.9 (Supplementary Table 2), while the single NiV Malaysia anti-G ELISA correlation was 0.809.

Additionally, the quantification of G-biosensor activation was compared to neutralization of NiV, lentivirus, Vesicular Stomatitis Virus (VSV) particles pseudotyped with the F and G of NiV, and recombinant Cedar virus bearing NiV-F and G (Supplementary Table 3). Significant correlations were only observed with both NiV Bangladesh and Malaysia, as well as all VSV pseudotypes. The highest correlation (r = 0.974) was observed with lab 15b’s neutralizing NiV Bangladesh results using the PRNT_50_ method. However, the correlation of other neutralizing assays with G-biosensor was generally lower or not significant when compared to all lentivirus pseudotypes and recombinant Cedar virus (r < 0.7497). These findings suggest that quantitative anti-G ELISA and G-biosensor measurements correlate with most NiV and VSV pseudotype neutralization assays. The exception was the lab 15b assay, where correlation appeared to be generally weaker than seen with anti-G ELISA results.

### Characterization of IgM and IgG clinical samples from Bangladesh

**Figure 3.**
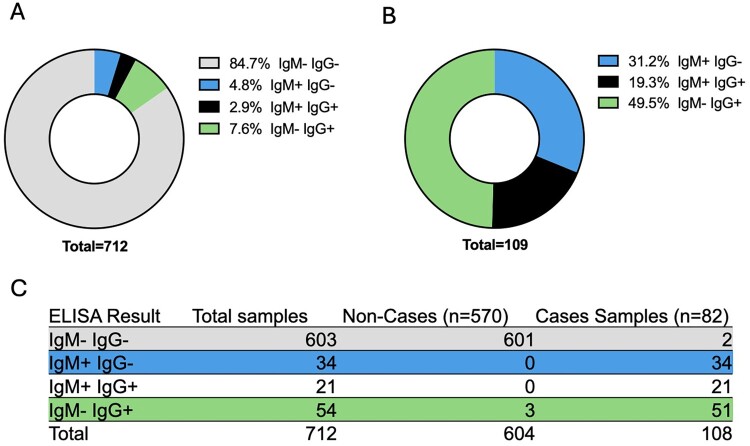
Anti-NiV IgM and IgG distribution in human clinical samples from Bangladesh. (A) Distribution of all samples tested classified according to IgM and IgG ELISA results. (B) Distribution of ELISA positive samples classified according to IgM and IgG ELISA results. (B) Samples number breakdown of all the samples tested from NiV confirmed cases and non-case.

Serological tests need thorough validation to precisely determine the level of past infections in human and animal hosts. Since 2001, NiV outbreaks have recurred on a near-annual basis in Bangladesh. Between 2012 and 2021, a total of 712 serum or plasma samples were collected from 82 NiV cases confirmed by RT – PCR and/or anti-NiV IgM ELISA and 581 non-cases (Figure 3). This collection represents a large and unique set of confirmed NiV cases, allowing us to reliably assess the performance of CDC IgG ELISA and G-biosensor. Since these samples were collected during the acute and covalence phase of the disease, 34 samples were IgM positive and IgG negative, 21 samples were found IgM positive and IgG negative and 54 samples IgG positive only. To accurately measure the sensitivity and specificity of CDC IgG ELISA, we restricted our analysis to IgM negative samples from confirmed cases (n = 54) that are typically collected several weeks after illness and non-cases (n = 601). In this IgM negative subset, 54 out 54 samples were found positive by the CDC IgG ELISA and 3 out of 601 non-cases samples were IgG positive. These data indicate that CDC-developed anti-NiV IgG ELISA had a sensitivity of 100% for detecting long-term NiV survivors, and a specificity of 99.5% since 3 out 604 samples from non-cases yielded a positive result. Therefore, the relative distribution of samples based on ELISA results was 31.2% IgM + and IgG-, 19.3% IgM + IgG+, and 49.5% IgM-IgG + (Figure 3B).

### Diagnostic performance of α NiV G mix-and-read assay compared to IgM and IgG ELISA

**Figure 4.**
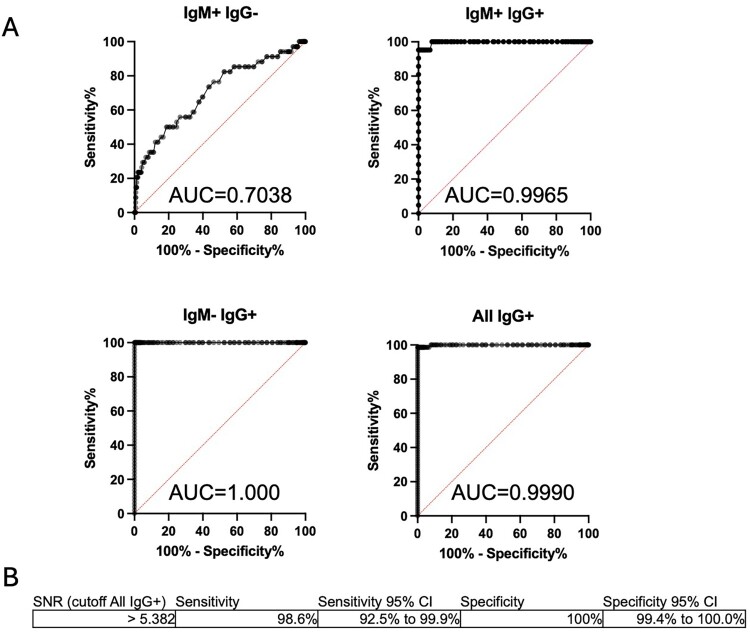
Receiver operating characteristics (ROC) curves and area under the curve (AUC) of ELISA-classified samples. ELISA results from non-cases and NiV-confirmed cases were used for ROC analyses. (A) αNiV-G mix-and-read (MR) assay performance was determined from the ROC analysis using samples from confirmed cases with IgG-positive results and from non-cases. (B) Anti-G NiV mix-read-assay cutoff determination was determine using ROC analysis of all ELISA IgG + samples from confirmed cases (n = 72) and non-cases (n = 604) from Bangladesh. Assay cutoff signal to noise ratio (SNR) of more than 5.38 is considered positive. Assay sensitivity and specificity was determined based on ROC and associated 95% Confidence Interval (CI) were calculated using Wilson/Brown method.

**Figure 5.**
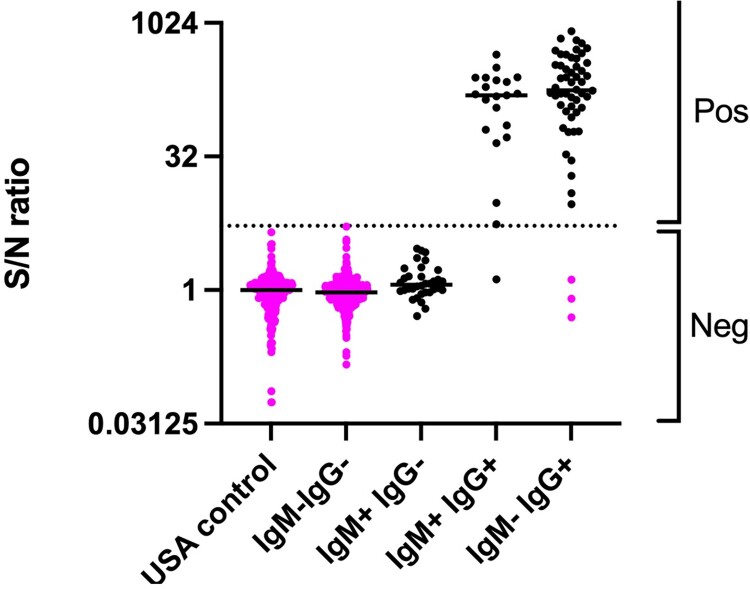
Distribution of αNiV-G MR assay signal classified by anti-NiV IgM and IgG ELISA results. Black dots represent SNR results from NiV-confirmed cases, and magenta dots, from non-cases. Dotted line indicates MR assay threshold.

**Figure 6.**
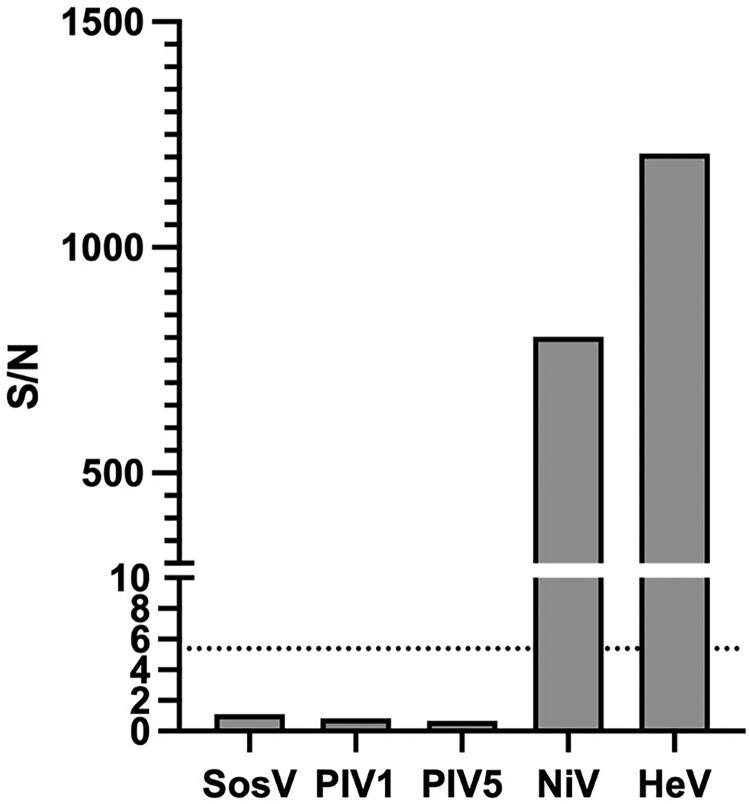
Assessment of αNiV-G MR assay cross-reactivity towards other paramyxoviruses. Mouse hyperimmune ascites from select paramyxoviruses were used in the αNiV-G MR assay. Dotted line indicates the MR assay threshold. SosV, Sosuga virus; PIV1, parainfluenza virus 1; PIV5, parainfluenza virus 5; HeV, Hendra virus.

The sample set characterized by ELISA was tested with the faster and simpler mix-and-read (MR) method using G-biosensor. The αNiV-G MR was performed by adding the G-biosensor solution to an equal volume of serum or plasma and incubating for 20 min before adding the NanoLuc substrate solution for 10 min and then reading the plate with a luminometer. SNRs were calculated by dividing the RLU from the experimental sample by the RLU of normal human serum control. The assay cutoff value was determined by ROC analyses based on CDC anti-NiV ELISA results (Figure 4). The area under the curve (AUC) was calculated as an indication that ROC correctly classified confirmed cases and non-cases. An AUC of 1 would represent a perfect classifier, while AUC of 0.5, indicates a random result. The ROC analysis curve of the IgM ^+ ^IgG^–^ subset had AUC = 0.704, indicating that the αNiV-G MR assay is not a reliable method for identifying NiV cases when only the IgM antibody response is detectable. In contrast, the IgM ^+ ^IgG^+^ subset was a near-perfect classifier (AUC = 0.997), and the IgM^-^IgG^+^ subset produced a perfect result (AUC = 1) (Figure 4). Based on these ROC analyses, the αNiV-G MR assay threshold was established to detect the maximum number of IgG^+^ samples while maintaining a high level of specificity. An ROC curve combining the results from the IgM ^+ ^IgG^+^ and IgM^-^IgG^+^ subsets was also generated (AUC = 0.999). Based on the latter curve, the αNiV-G MR assay SNR positive threshold was established at 5.38, which allowed the detection of IgG^+^ NiV cases with a sensitivity of 98.6% and specificity of 100%. A total of 300 additional serum samples from the USA was subjected to testing to compare MR data to sera from NiV non-endemic country. Using this assay threshold value, all IgG^+^ samples from confirmed cases and non-case samples were precisely grouped except for one false negative from IgM ^+ ^IgG^+^ sample set (Figure 5). Antibodies raised against NiV-G and HeV-G can significantly cross-react [26,27]. The potential of anti-HeV antibodies and antibodies against other paramyxoviruses (Sosuga virus, parainfluenza virus 1, and parainfluenza virus 5) for activating the G-biosensor was tested using mouse hyperimmune ascites. Only anti-NiV and anti-HeV antibodies strongly activated the G-biosensor (Figure 6). Together, these data demonstrate that the αNiV-G MR assay is faster than ELISA and accurately identify individuals who had survived NiV infection but failed to detect anti-NiV IgM-positive samples lacking anti-NiV IgG; anti-HeV antibodies also strongly activated αNiV-G biosensor.

### Development and persistence of IgG and αNiV-G MR positivity in survivors

**Figure 7.**
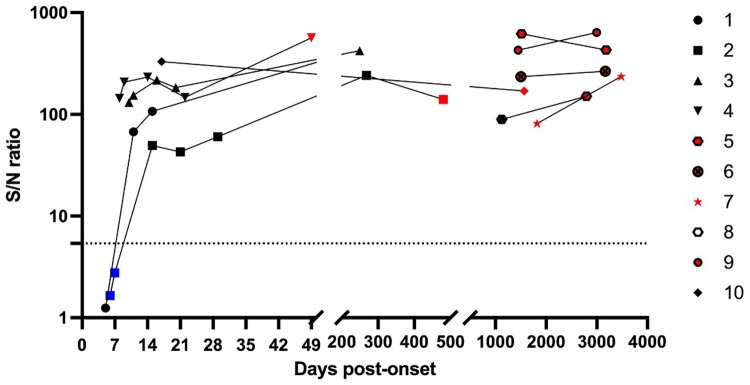
Detection of antibody responses over time in NiV survivors. αNiV-G MR assay was used with samples from NiV survivors from whom multiple samples were available. Each symbol represents SNR for the same patient over time. Dotted line represent assay cutoff. Colors are used to indicate ELISA results: blue, IgM ^+ ^IgG^-^; black, IgM ^+ ^IgG^+^; red = IgG + IgM-

Our sample set consisted of 10 Nipah disease survivors from Bangladesh from whom multiple samples were obtained over time. To estimate the duration needed to obtain a positive result in NiV-infected individuals, we examined the SNR (Figure 7) produced by samples from the early, recovery, and convalescent stages of illness. Up to 8 days after symptom onset, all 3 samples from patients #1 and #2 tested negative with the αNiV-G MR assay but all were IgM positive; 1 of 3 samples from patient #1 was also IgG positive. This single IgM ^+ ^IgG^+^ sample from patient #1 represents the only IgG^+^ sample that was not detected by the αNiV-G MR assay; samples collected from the same patient 5 days later (11 days post symptom onset) all tested positive. In contrast, all samples taken more than 7 days post symptom onset were positive. In 3 individuals (patients #2, #3, and #8), IgM was still present for a minimum of 249 days after the onset of symptoms, with patient #8 having detectable IgM for over 3 years. Overall, SNR generally increased over time and did not show any important signs of decreasing even after more than 8 years, with neutralizing titers ranging from 80 to over 5120 (Supplementary Table 4). These findings suggest that the αNiV-G MR assay is expected to yield positive results ∼8–15 days after symptom onset, and that this signal should last multiple years.

### Detection of anti-NiV antibodies in animal hosts

**Figure 8.**
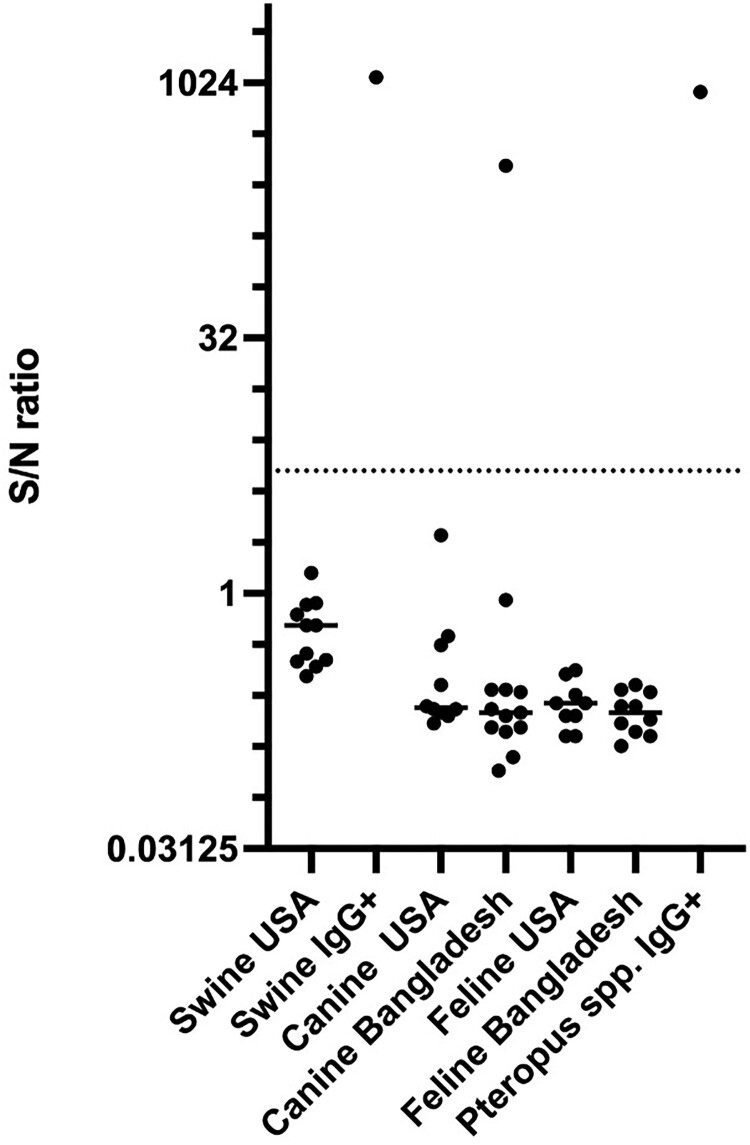
Detection of NiV antibodies in animal samples. αNiV-G MR was used with serum samples from animals from Bangladesh, Malaysia, and USA. αNiV-G MR negative control samples are from the USA. MR-positive swine, dog, and *Pteropus* bat sample pools were previously found positive by ELISA.

NiV can infect various wild and domestic animal species, including pigs, which served as the amplifying host during the initial NiV outbreak in Malaysia. In contrast to ELISA, the detection of antibodies with the αNiV-G MR assay does not require species-specific reagents like secondary antibody conjugates. The potential of utilizing the αNiV-G MR assay in animal species was tested using the protocol validated for human samples. Sera from pigs, cats, and dogs from the USA, a non-NiV-endemic region, were included as negative controls. Due to the limited number of animal samples available from NiV-endemic countries, we conducted tests on only one pig that was naturally infected during the NiV outbreak in Malaysia, a pool of IgG-positive sera from *Pteropus* bats and sera from cats and dogs in Bangladesh. A dog sample from Bangladesh, along with the bat and Malaysian pig samples with known anti-NiV antibodies, exhibited strong SNR compared to the control animals from the USA (Figure 8).

## Discussion

Our study shows the development, optimization, and validation of a split NanoLuc luciferase biosensor for accurately identifying NiV cases from a large cohort of patients from Bangladesh. The performance of this simpler and faster αNiV G MR was comparable in sensitivity and specificity to CDC’s anti-NiV IgG ELISA, making it ideal for retrospective identification of cases and tracking anti-G antibody levels over time.

Diagnosis of acute NiV infections is performed using RT–PCR or anti-NiV IgM ELISA [28]. The αNiV-G MR assay failed to reliably detect IgM only positive samples as indicated by the poor AUC value of 0.7. Therefore, anti-NiV IgM in samples lacking anti-NiV IgG cannot be used to diagnose acute NiV cases early during the disease. It is unclear why anti-NiV G IgM detection is poor or undetectable by the G biosensor. One possible explanation for this observation is that IgM generally has lower affinity than IgG. Alternately, its structure does not productively complement NanoLuc fragments due to the angle differences at which pentameric IgM structure could result in increased steric hinderance compared to the dimeric IgG when binding to the G biosensor. However, further studies are necessary to precisely identify the underlying cause for the undetectable or insufficient activation of G biosensor by IgM early in the infection, and to ascertain whether this observation is general or specific to the NiV G antigen or biosensor design used.

G mediates NiV binding to its cellular receptors ephrin B2 and B3 [29]; therefore, the αNiV-G biosensor contains the receptor binding domain, a key region target of neutralizing antibodies against NiV and HeV [30–33]. The fusion glycoprotein F is also a major target of neutralizing antibodies [34,35]. Strong correlation levels were observed between the αNiV-G MR assay and quantitative anti-NiV G IgG ELISA tests using WHO NiV standard samples conducted by various laboratories [19]. This indicates high level of agreement with the MR assay, so similar results are expected from both ELISA and MR, as we previously reported for the SARS-CoV2 RBD MR assay [15]. Despite using NiV-infected cells, CDC IgG ELISA results showed almost perfect agreement, with only 1 false negative result out of 82 for the MR assay and 3 false positives out of 603 for CDC IgG ELISA. Although significant correlation was observed with several NiV neutralization assays (Supplementary Table 3), this lower level of correlation might be explained by the probable presence of anti-F neutralizing antibodies that would not be detected by the αNiV-G MR assay.

Our research also offers valuable insights into the kinetics of anti-NiV antibody development among survivors. As shown in Figure 7, all samples were identified as positive later than 7 days post symptom onset. Thus, we expect that the αNiV-G MR assay would yield positive results around 1–2 weeks after the onset of symptoms, corresponding with the time of rising anti-NiV IgG. Two weeks after symptom onset, SNR levels remained similar for a minimum of 8 years in all the 5 patients examined. The same patients’ samples also exhibited neutralizing antibodies for several years (Supplementary Table 4). According to these findings, we anticipate that the αNiV-G MR assay will likely identify cases a few weeks after the date of symptom onset and as late as many years after, validating the αNiV-G MR assay as a valuable tool for retrospectively identifying NiV cases. This faster, easier assay should greatly facilitate surveillance efforts. HeV and NiV share a high level of sequence homology, enough that CDC anti-HeV ELISA was initially used to identify NiV infection in the outbreak in Malaysia [3]. As expected, anti-HeV antibodies strongly activated the G biosensor, suggesting that additional tests would be required to differentiate the anti-NiV G antibody response from anti-HeV G. This was previously achieved by comparing anti-G ELISA or Bio-Plex protein array results using soluble NiV and HeV G, as the antigen homologous antibody response was higher in all the specimens tested [36,37]. Developing an anti-HeV-G biosensor might also allow differentiation between NiV and HeV infections using a combination of MR assays. Alternatively, αNiV-G MR assay positive results could be confirmed by performing HeV and NiV neutralization tests [37]. Furthermore, the high level of cross-reactivity of αNiV-G biosensor (strain Malaysia) with HeV serum suggests that developing other biosensors using G sequences from more closely related NiV strains is unlikely to significantly affect the mix-and-read assay performance.

NiV circulates in animal hosts until spillover events result in human infections and, occasionally, to further spread of the virus by human-to-human transmission. In Bangladesh, infections have been linked to the consumption of raw date palm sap contaminated by *Pteropus* bats [38]. Based on the αNiV-G MR assay principle (Figure 1), anti-NiV antibodies are expected to be detected independently of the animal origin, which is advantageous for performing ecological studies, outbreak investigations, and enhancing surveillance in animals. In suppport of the species independence of the MR assay, our data indicated that both rabbit and human anti-G monoclonal antibodies, such as nAH1.3, resulted in similar SNR (Figure 2). In addition, positive sera from dogs, bats, and pigs were correctly identified, suggesting that the assay could be readily adapted and validated to various animal species. Given the ease of use and proven performance of the αNiV-G MR assay in humans, we expect the use of this technology to be expanded to animal samples, as demonstrated for SARS-CoV2 [39–42].

The versatility of split NanoLuc technology extends beyond anti-NiV antibody detection. Sensitive diagnostic tools for antigen and nucleic acid detection can be adapted to point-of-care diagnostic by lyophilizing thermostable reagents in a one-pot reaction format [13,17], using low-cost handheld luminometers recently developed and deployed in Bangladesh to detect anti-SARS-CoV2 antibodies [43]. The extensive validation and robust performance of this technology for NiV demonstrate its utility and advancement as a valuable diagnostics platform.

## Acknowledgements

We thank Tatyana Klimova for assistance editing the manuscript and Laura Morgan for providing technical assistance. The findings and conclusions in this report are those of the authors and do not necessarily represent the official position of the Centers for Disease Control and Prevention.

## Supplementary Material

SlideS1.jpeg

SlideS2.jpeg

SlideS4.jpeg

SlideS3.jpeg
