# Supplementary figures and images for: Streamlined detection of Nipah virus antibodies using a split NanoLuc biosensor
Source: Emerg Microbes Infect. 2024 Aug 28;13(1):2398640. doi: 10.1080/22221751.2024.2398640 (PMC11391874; doi:10.1080/22221751.2024.2398640)

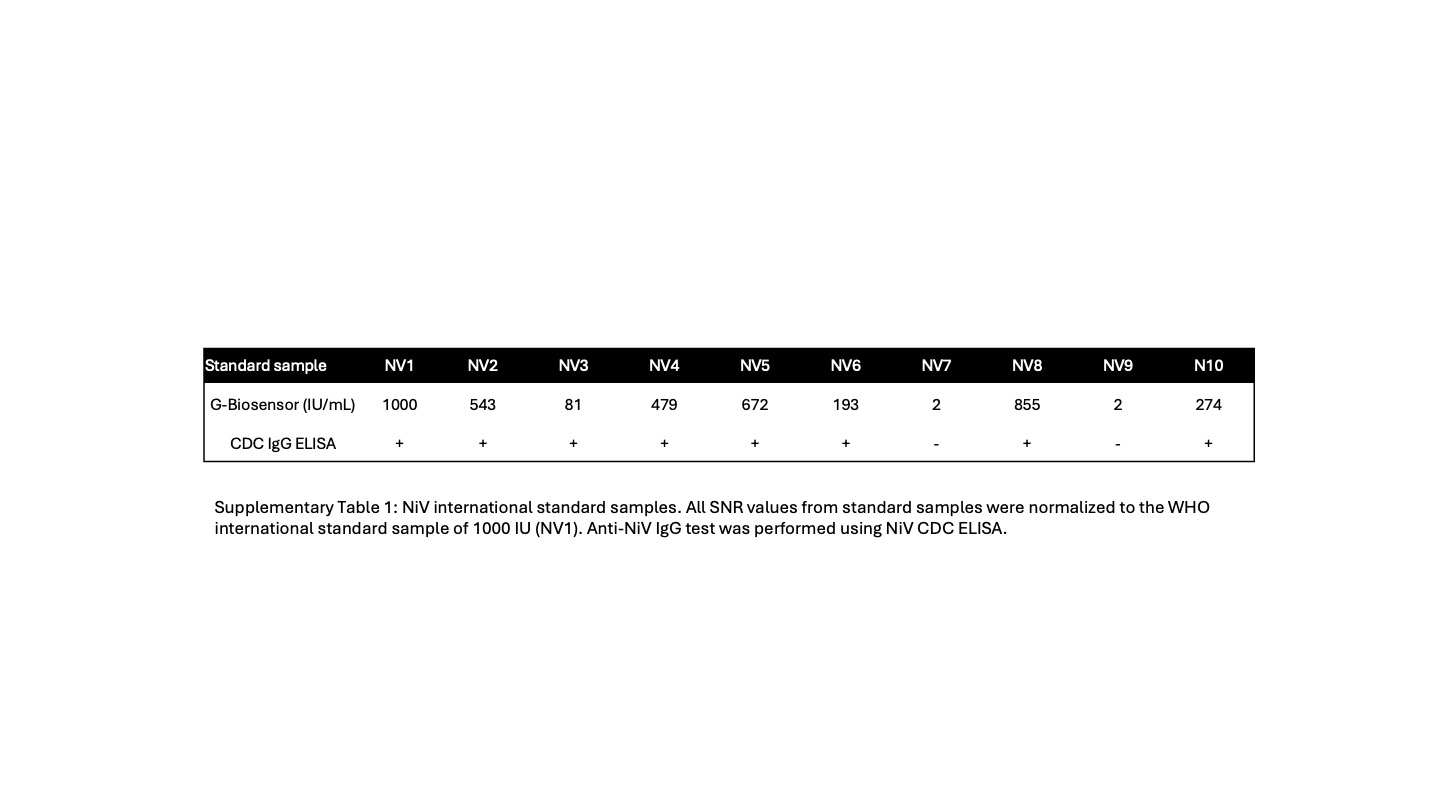

Supplement: SlideS1.jpeg [file TEMI_A_2398640_SM0121.jpeg]

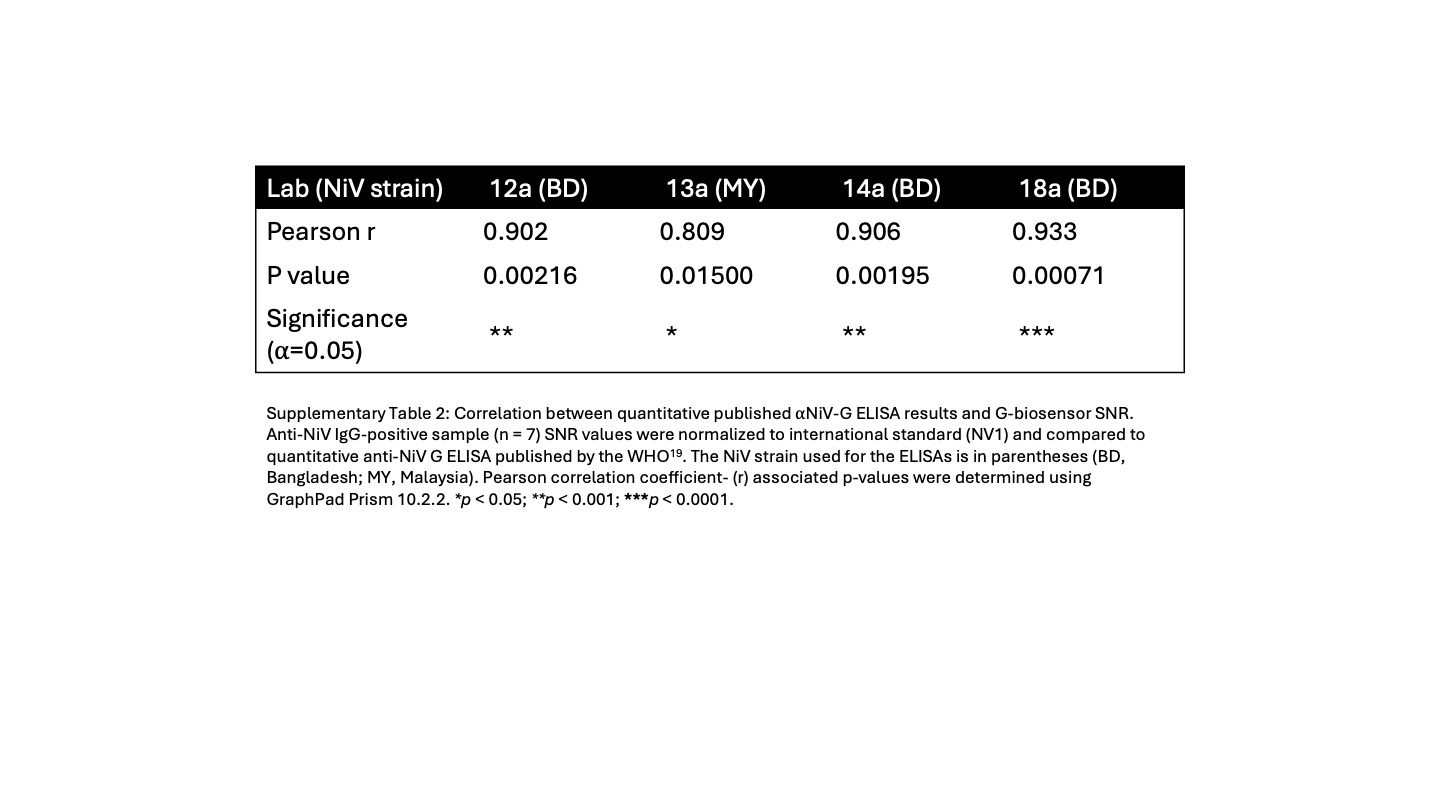

Supplement: SlideS2.jpeg [file TEMI_A_2398640_SM0120.jpeg]

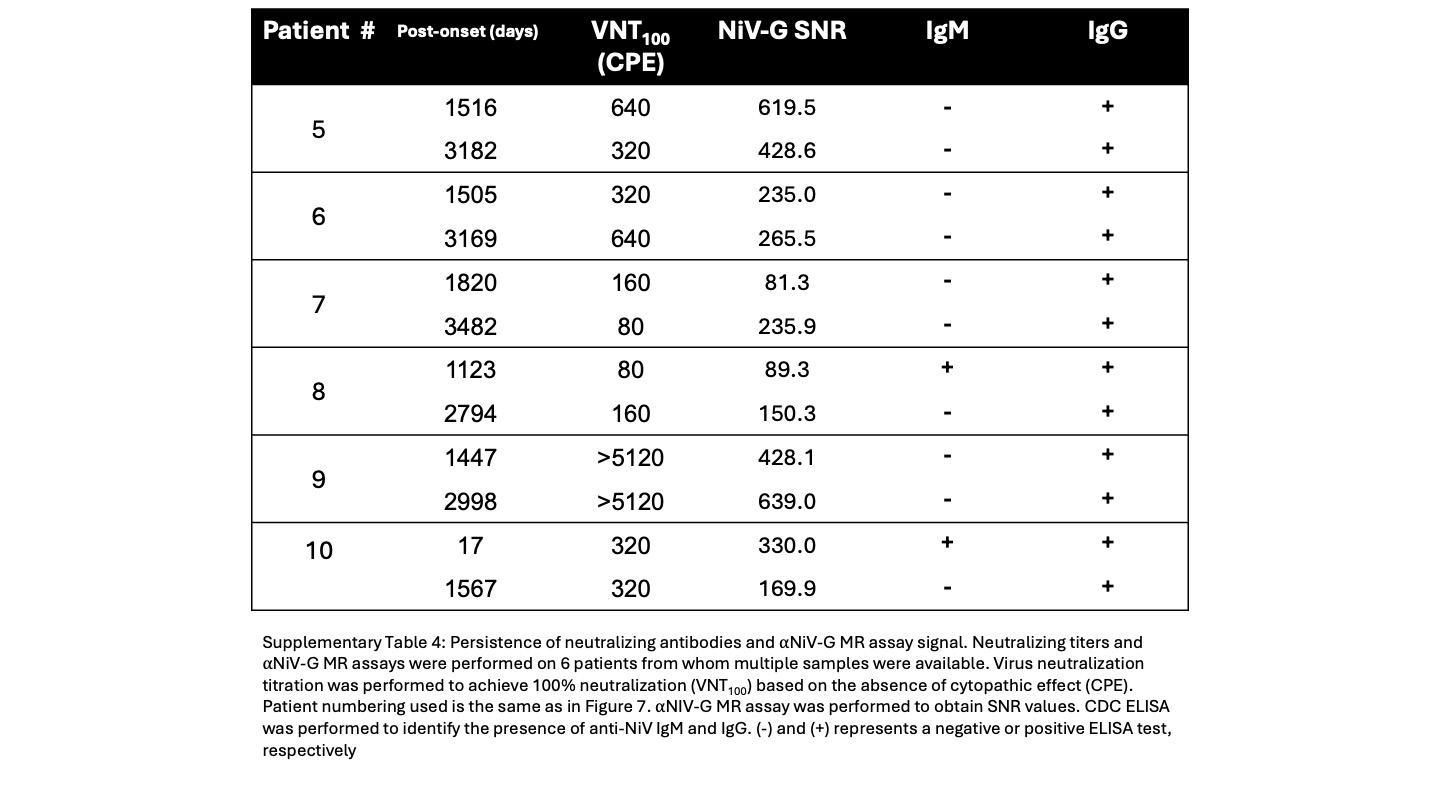

Supplement: SlideS4.jpeg [file TEMI_A_2398640_SM0119.jpeg]

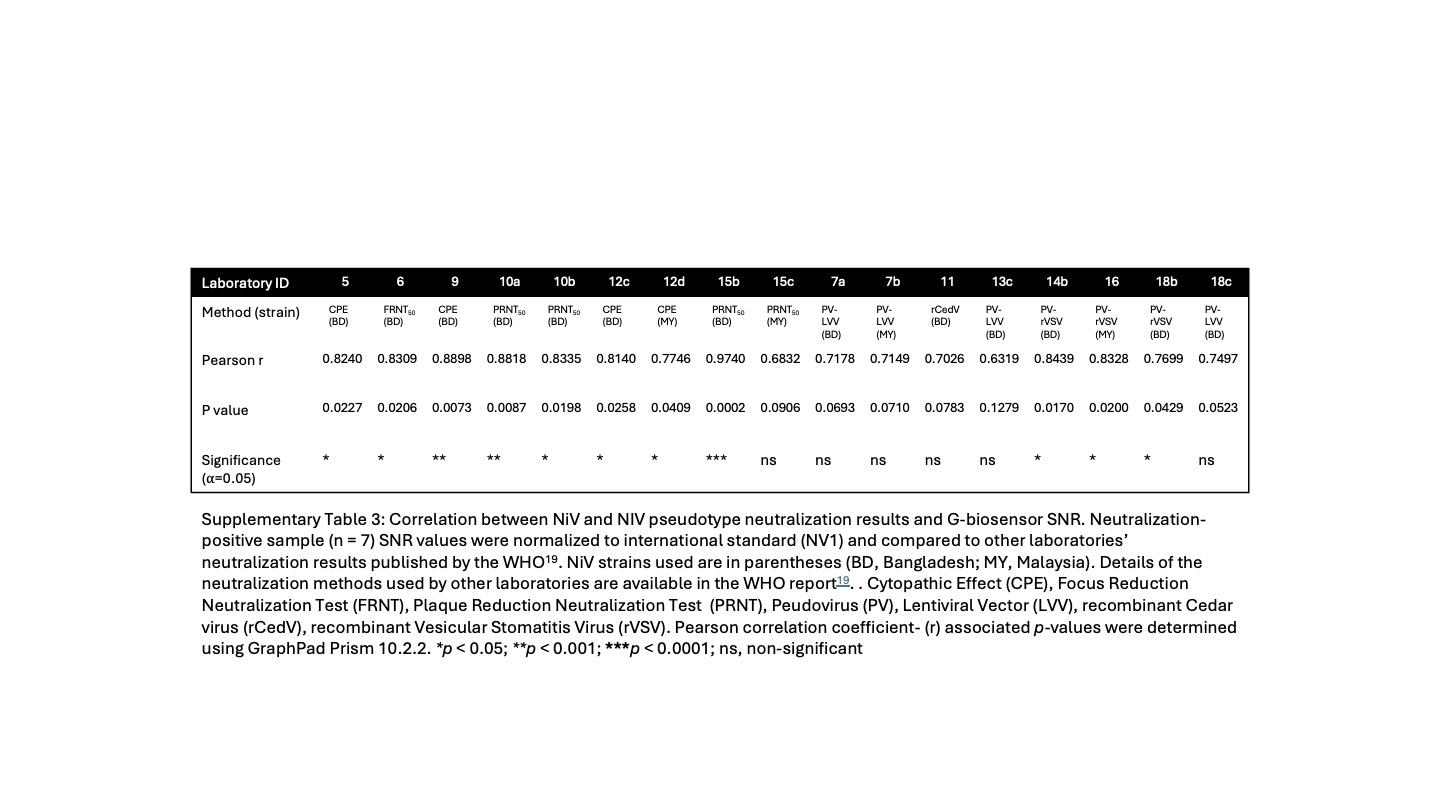

Supplement: SlideS3.jpeg [file TEMI_A_2398640_SM0118.jpeg]
